# Spontaneous Neutrophil Migration Patterns during Sepsis after Major Burns

**DOI:** 10.1371/journal.pone.0114509

**Published:** 2014-12-09

**Authors:** Caroline N. Jones, Molly Moore, Laurie Dimisko, Andrew Alexander, Amir Ibrahim, Bryan A. Hassell, H. Shaw Warren, Ronald G. Tompkins, Shawn P. Fagan, Daniel Irimia

**Affiliations:** 1 Surgery Department, Massachusetts General Hospital and Harvard Medical School, Boston, Massachusetts, United States of America; 2 BioMEMS Resource Center, Center for Engineering in Medicine and Surgical Services, Boston, Massachusetts, United States of America; 3 Shriners Hospital for Children, Boston, Massachusetts, United States of America; 4 Departments of Pediatrics and Medicine, Infectious Disease Unit, Massachusetts General Hospital and Harvard Medical School, Boston, Massachusetts, United States of America; University of Florida College of Medicine, United States of America

## Abstract

Finely tuned to respond quickly to infections, neutrophils have amazing abilities to migrate fast and efficiently towards sites of infection and inflammation. Although neutrophils ability to migrate is perturbed in patients after major burns, no correlations have yet been demonstrated between altered migration and higher rate of infections and sepsis in these patients when compared to healthy individuals. To probe if such correlations exist, we designed microfluidic devices to quantify the neutrophil migration phenotype with high precision. Inside these devices, moving neutrophils are confined in channels smaller than the neutrophils and forced to make directional decisions at bifurcations and around posts. We employed these devices to quantify neutrophil migration across 18 independent parameters in 74 blood samples from 13 patients with major burns and 3 healthy subjects. Blinded, retrospective analysis of clinical data and neutrophil migration parameters revealed that neutrophils isolated from blood samples collected during sepsis migrate spontaneously inside the microfluidic channels. The spontaneous neutrophil migration is a unique phenotype, typical for patients with major burns during sepsis and often observed one or two days before the diagnosis of sepsis is confirmed. The spontaneous neutrophil migration phenotype is rare in patients with major burns in the absence of sepsis, and is not encountered in healthy individuals. Our findings warrant further studies of neutrophils and their utility for early diagnosing and monitoring sepsis in patients after major burns.

## Introduction

Today, sepsis is the leading cause of death after major burn injury [1], [2] and a major cause of death in the intensive care units, with a mortality rate of about 30% [3]. Identifying sepsis early is critical, considering that for every 6 hours delay in the diagnosis of sepsis, survival decreases by 10% [4]. However, identifying sepsis in burn patients is challenging, because it is masked by the systemic inflammation response syndrome (SIRS), which occurs in almost all patients with major burns [5]. Diagnosis may also be delayed by the time required for microbiological confirmation of infection. Although advanced mass spectrometry tools in microbiology labs can accelerate the identification of infections in blood, they still require at least 12–24 hours of blood culture before the bacteria reach levels that can be detected [6], [7]. In the absence of sepsis, inappropriate use of antibiotics can have long term consequences for patients by interfering with the normal microbiome, facilitating the development of multidrug resistant bacterial strains, and increasing the cost of hospitalization [8]. Contributing to the challenge of diagnosing sepsis is the fact that the pathophysiology of sepsis is not well understood and there is no reliable marker for sepsis. For example, C-reactive protein (CRP) is a marker of the inflammatory response post-burn and recombinant-CRP has been shown to help treating sepsis [9]. However, the levels of CRP fail to predict infection or sepsis in patients with major burns [10]. Neutrophil CD64 expression can be markedly up-regulated at the onset of bacterial infections. However, recent studies have found that CD64 also increases after major trauma and SIRS [11] or sterile insult after major surgery [12]. Numerous other molecules have been considered as potential markers, including IL1, IL6, procalcitonin, and reactive oxygen species. However, none has yet been proven effective in the clinic [13]–[15].

One opportunity for detecting sepsis in patients with major burns arises from the study of innate immune responses, particularly those of neutrophils, the major white blood cell population, and first responders to tissue injury. Among the features of the complex neutrophil phenotype, chemotaxis could be an appropriate integrator and sensitive measure for neutrophil function. Neutrophils can integrate a broad range of chemokine and metabolic changes triggered by the burn injury [16]. Chemotaxis is altered well before other important neutrophil functions are affected, including phagocytosis [17] and the production of reactive oxygen species [18], cytokines [19] or release of lytic enzymes [20]. However, until now, neutrophil chemotaxis changes measured in burn patients have been nonspecific and no features specific to sepsis have been identified. Earlier efforts to characterize neutrophil chemotaxis in sepsis, using traditional cell migration tools, such as the transwell assay [21], have been hampered by intrinsic limitations of these technologies, such as the instability of the chemical gradients, and lack of single cell resolution. First employed for studies of cell migration a decade ago [22], microfluidic assays enabled very precise control of the shape and stability of chemical gradients during neutrophil chemotaxis [23]–[27]. However, biological issues related to the intrinsic variability of directionality and speed of neutrophils moving on flat surfaces limited the precision of the motility phenotype measurements in these devices [28], [29]. Recently, microfluidic devices that confine the moving cells into micro-channels enabled us to decouple the measurement of speed from directionality and persistence and to independently quantify these parameters with high precision [30], [31].

Here, we performed comprehensive measurements of neutrophil migration in blood samples from adult patients after major burns (>20% total body surface area). We monitored the changes in neutrophil migration using microfluidic devices for up to one month after injury, across 18 independent parameters, in 74 blood samples from burn patients and healthy subjects. Blinded, retrospective analysis determined the presence of sepsis in the group of patients in this study. By studying the correlations between neutrophil migration and sepsis, we identified one unanticipated neutrophil migration phenotype that was highly specific for sepsis. We found that spontaneous migration of neutrophils in the absence of a chemoattractant associated with sepsis could begin before sepsis is diagnosed, and ceased after sepsis was effectively treated. These results could have practical implications for the diagnosis and monitoring of sepsis in patients after major burns, as well as warrant further studies to better understand the roles of neutrophils during sepsis.

## Materials and Methods

### Design and fabrication of the microfluidic devices

The design of the device enables one to generate chemical gradients in two simple steps performed at the beginning of the experiment, after which the device does not require any user intervention or external syringe pumps to operate. The device is first primed with chemoattractant and then washed with a buffer solution, based on principles described in detail before [32]. A gradient forms between an array of sources of chemoattractant and a large buffer reservoir, connected through long channels with small cross section. An array of orthogonal side-channels (6 µm width, 3 µm height), primed with the chemoattractant, serve as source and a central channel (500 µm width, 50 µm height), washed with media, serves as a sink. The array of channels incorporates straight channels for measuring the speed of neutrophil migration, as well as channels with several types of mazes (centered rectangle, off-centered rectangle, and posts) to quantify neutrophil directionality and persistence during migration.

The microfluidic devices were manufactured using standard techniques. Two layers of photoresist (SU8, Microchem, Newton, MA), the first one 3 µm thin and the second one 50 µm thick, were patterned on one silicon wafer by sequentially employing two photolithography masks and processing cycles according to the instructions from the manufacturer. The wafer with patterned photoresist was used as a mold to produce PDMS (Polydimethylsiloxane, Fisher Scientific, Fair Lawn, NJ) parts, which were then bonded irreversibly to standard glass slides (1×3 inches, Fisher).

### Blood samples

Blood samples of 3 mL were drawn from patients with large thermal injuries admitted to the Massachusetts General Hospital (MGH). Enrolment criteria included burns covering at least 20% of total body surface area, age between 18 and 81, and we enrolled both male and female, proportionally distributed among ethnic groups. The first sample was obtained within 72 hours after burn injury, and two more samples were drawn at 48 hour intervals afterward (phase 1). Samples were then obtained weekly for up to two months during treatment (phase 2). During a suspected septic event, three additional samples were drawn at 48 hour intervals (phase 3). No samples were collected within 24 hours after an operative procedure.

### Ethics Statement

All patient samples were obtained with written informed consent and through procedures approved by the Institutional Review Board at the Massachusetts General Hospital (2008-P-002123).

### Neutrophil isolation

Using sterile technique, neutrophils were isolated from whole blood by density gradient separation using HetaSep for 15 min. at room temperature. Neutrophils were then purified using the EasySep Human Neutrophil Kit (STEMCELL Technologies Inc. Vancouver, Canada) following the manufacturers protocol. The purity of neutrophils was assessed to be>98% using a Cell Counter. The final aliquot of neutrophils was re-suspended in 20–50 µL of 1× HBSS +0.2% HSA before loading the neutrophils into the chemotaxis device. Samples were processed within one hour after each blood draw, and were maintained at 37°C.

### Neutrophil chemotaxis measurements

Chemokine concentration gradients are maintained for at least 3 hours in the axial direction of the channels by diffusion between the dead-end channels (primed with chemokine at the start of the experiment and functioning as source) and the neutrophil loading channel (washed with buffer when neutrophil suspension is loaded, the and functioning as a sink). Approximately 15 minutes before neutrophils were loaded, the microfluidic device was primed with the chemokine formyl-methionyl-leucyl-phenylalanine (fMLP - 100 nM), leukotriene B_4_ (LTB_4_ - 100 nM), or no chemoattractant, serum-free Hank's Balanced Salt Solution (HBSS) with 0.2% Human serum albumine (HSA). All conditions included extracellular matrix protein fibronectin [100 µM]. The side channels are closed at the distal end, devices are primed with chemoattractant and a linear gradient is formed along the length of the channel when the central well is washed with media. For this, a 1 mL syringe filled with the solution of chemoattractant and fibronectin was connected to one port of the device, and the outlet port was blocked with a hemostat. By applying pressure to the syringe, the solution was instilled into the device, and the displaced air diffused out through the PDMS. Neutrophils were then infused into the device and allowed to settle in the main channel by clamping the ports of the device. Neutrophil migration in the direction of chemoattractant started immediately, and was recorded using and time-lapse imaging on a Biostation IM at 37C and 80% humidity. Images of the moving neutrophils are acquired automatically every 2 minutes for up to 2 hours, from at least 30 distinct locations on each microfluidic device (minimum 10 per condition).

The analysis of neutrophil migration was performed by an independent researcher with no information about the status of the patients. All migrating neutrophils were manually tracked in each sample, and velocities were calculated using Image J (NIH). Because the moving neutrophils are mechanically confined in the 6 µm width, 3 µm height channels, neutrophil migration paths are constrained and cell trajectories are uncomplicated to measure.

### Sepsis criteria

For the purpose of this study, sepsis was established blindly and retrospectively, by a physician not involved in the care of the patients in this study. Sepsis was defined based on the presence simultaneously of documented infection and SIRS, following the guidelines from the American College of Chest Physicians (ACCP), Society of Critical Care Medicine (SCCM) and the European Society of Intensive Care Medicine (ESICM) [5]. SIRS was established based on two or more of the following criteria: temperature above 38°C or below 36°C, tachycardia (heart rate above 90 beats per minute), tachypnea (respiratory rate faster than 20 breaths per minute), and white blood count above 12×10^9^/L or below 4×10^9^/L or more than 10% immature (band) forms. Infections were established based on documented positive cultures of blood samples, intra-operative deep wound specimens, deep tracheal aspirate, or urine samples. Patients who had positively documented cultures without signs of SIRS were not considered septic. Septic patients were considered no longer septic when cultures converted into sterility after antimicrobial treatment. Antimicrobial treatment was not considered as an associated factor for diagnosis of infection due to frequent empirical antimicrobial regimens for common SIRS conditions in burn patients. Thus, our criteria for sepsis were stricter than the current guidelines that consider suspected infections [5].

### Statistical Analysis

STATA software (StataCorp., College Station, TX) was utilized to apply a logistic model to the entirety of our neutrophil functional data set and determine whether a patient's septic status could be predicted by their neutrophil phenotypic function. Exploratory analysis determined 3 variables, all quantifying the spontaneous migration in the absence of chemoattractant, to significantly correlate to a septic diagnosis in the clinic. These three variables included: number of neutrophils migrating in the absence of chemoattractant (NMC_N_), oscillatory migration in the absence of chemoattractant (OM_N_) and the total length of migration in the channels in the absence of chemoattractant (TL_N_). A neutrophil activation score in the absence of chemoattractant (NAS_N_) was defined as NMC_N*_(TL_N_ + OM_N_). A logistic model (Generalized Estimating Equations (GEE)) was used to determine statistical significance of the NAS_N_ variable as a clinical biomarker for sepsis. The GEE took into account the patient's repeated NAS_N_ measurements over time and did not require normality of the predictor variable. To test if the mean of the activation state during infection is different than that of the infection-free state, we used a random intercept linear mixed effects model (repeated measures of ANOVA). To compare the parameters of neutrophil migration between healthy controls and burn patients we used a t-test and difference s were considered significant for p<0.05.

## Results

### Microfluidic platform for probing neutrophil migration phenotype

The goal of this study was to measure changes of the neutrophil migration phenotype in patients with major burns and sepsis and to identify specific changes associated exclusively with sepsis. The study was enabled by microfluidic devices designed for precision measurements of the neutrophil migration phenotype. Two critical features contributing to the precision of the measurements are the confinement of moving neutrophils in channels with cross-sections smaller than the neutrophil size, and the placement of bifurcations and post-obstacles along the channels. In the straight section of the channels, we can measure neutrophil migration speed and persistence with high precision. At the posts and bifurcations, we force the moving neutrophils to make binary directional decisions, which are easy to quantify and compare.

**Figure 1:**
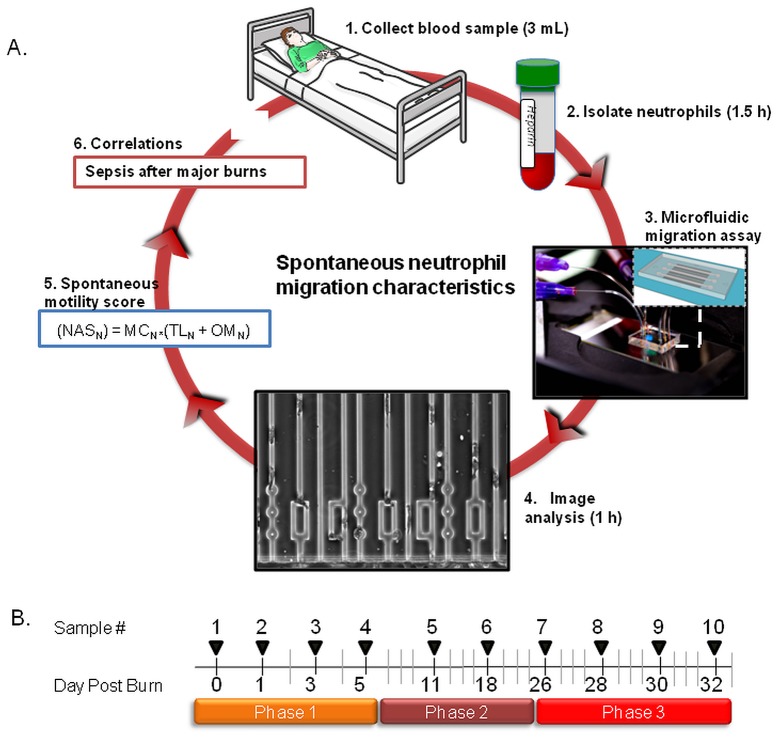
Measuring neutrophil migration in burn patients during sepsis. (A) Overview of the protocol using microfluidic devices to measure neutrophil migration in patients with major burns. (1) Three milliliters blood samples are obtained from patients with major burns. (2) Neutrophils are separated from blood using negative selection beads. (3) Microfluidic devices are primed with chemoattractant solutions and patient neutrophils are introduced into the neutrophil loading channel. (4) Neutrophils are observed using time-lapse imaging during migration through 3×6 µm channels and mazes of channels. (5) Images of moving neutrophils are recorded at single cells resolution and analyzed to quantify 18 neutrophil migratory parameters. (6) A neutrophil spontaneous motility score is calculated for each sample. (7) Correlations are tested between the Neutrophil activation score (NAS_N_) and sepsis in patients with major burns. This cycle is repeated every two days or less often for the duration of burn patient treatment, up to one month. (B) Typical timeline for sample collection from burn patients. During the first phase, samples are collected every two days for one week. A second phase continues with one sample every week for up to one month. A third phase is triggered by the clinicians when there is request for blood cultures, signs of fever, leukocytosis and/or thrombocytosis. During Phase three, samples are collected every two days for a week.

Neutrophils separated from patients' blood (**Fig. 1A** – steps 1 & 2) are loaded inside the device and their migration responses tested in 3 distinct conditions which include serum-free Hank's Balanced Salt Solution (HBSS) and gradients of formyl-methionyl-leucyl-phenylalanine (fMLP) and leukotriene B_4_ (LTB_4_) (**Fig. 1A** – step 3). The migration of neutrophils, recorded using automated microscopy, is analyzed for 6 parameters in each of the three conditions (**Fig. 1A** – step 4). While the neutrophils from healthy volunteers are able to migrate efficiently around the posts in the channel (**S1 Movie**), patient neutrophils often get stuck on the post, lose directionality, and exit the device (**Fig. 1A** – step 5, **S2 Movie**). All healthy neutrophils are able to pick the shortest path and follow the steeper gradient in the offset rectangle, whereas a large fraction of patient neutrophils loop inside the rectangle, unable to correctly follow the direction of the chemoattractant gradient. We then compared the neutrophil migration parameters to identify markers and scores to distinguish between burn patients with and without sepsis (**Fig. 1A** – steps 6 & 7). The analysis of neutrophil migration phenotype can be completed in less than 4 hours after the blood samples become available. Blood samples were collected every two days for the first 5 days in the hospital (phase 1) and once a week for three more weeks (phase 2). Additional samples were collected every two days for one week whenever the clinical team requested blood cultures from burn patients with fever and leukocytosis, and/or thrombocytosis (phase 3 - **Fig. 1B**).

### Neutrophil migration phenotype in patients with major burns

**Figure 2:**
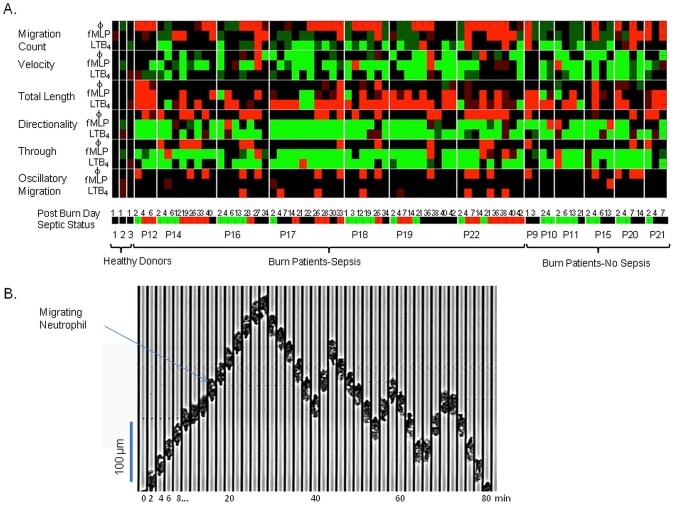
Neutrophil migration parameters in burn patients. (A) Heat-map showing the results of 18 neutrophil migratory phenotype measurements in 74 blood samples from 13 burn patients and three healthy volunteers. Specific measurements are explained in detail in Table 2, including averages for each parameter. Each parameter was measured in three conditions: in the absence of chemoattractant, and in the presence of fMLP and LTB_4_ chemoattractant gradients. Bright green illustrates 2 S.D. below healthy donor averages, light green illustrates 1 S.D. below healthy donor averages, bright red illustrates 2 S.D. above healthy donor averages, and light red illustrates 1 S.D. above healthy donor averages. Below the heat map, a color coded bar represents the status of the blood donor at the time of the draw. Red illustrates sepsis, green illustrates SIRS, and black illustrates no SIRS status. The day post burn is indicated above the bar, the patient identifier is presented below the bar. Healthy donors, burn patients with sepsis, and burn patients without sepsis are grouped by the accolades. (B) Kymograph showing an example of oscillatory migration of a neutrophil from a septic burn-patient, in the absence of chemoattractant. The time interval between successive frames is 2 minutes. Vertical scale bar is 100 µm.

We measured eighteen independent parameters of neutrophil migration in 74 samples from 13 patients with major burns, during treatment at Massachusetts General Hospital (**Table 1**) and 3 healthy volunteers. We validated first the device and compared it with previous studies [32], by measuring neutrophil migration in three healthy volunteers. The measured differences in migration speed between patients with major burns and healthy subjects are in agreement with previous observations [16], [32]–[39]. We also measured differences in the parameters of directionality and persistence between the neutrophils from patients with major burns and healthy controls. These differences between neutrophils from burn patients and healthy controls are summarized in **Table 2** and a heat map (**Fig. 2B**). Neutrophils from patients with major burns migrate in smaller numbers (MC), at slower average velocity (v), and are less directional (D). Fewer neutrophils reach the end of the channels in response to the two chemoattractants tested (T, TL). For the majority of samples, these parameters were on average at least two standard deviations below healthy donors.

**Table 1:** Summary of burn patient enrollment and hospitalization statistics.

Parameter	Value
Total number of patients	13
Total blood samples	71
Average size burn (TBSA)	39.3%
Patients with sepsis during hospital treatment	7
Patients with SIRS during hospital treatment	12
Patients with infection	7
Patients treated with antibiotics	13
Mortality	1

**Table 2:** Summary of neutrophil phenotypic migration parameters to an array of chemoattractants (no chemoattractant, fMLP, and LTB_4_).

Neutrophil Chemotaxis Parameter	Abbr.	Definition	Chemo-attractant	Healthy Donor (Avg. +/− Std. Dev.)	Burn Patient (Avg. +/− Std. Dev.)
1. Migration Count	**MC**	The total number of neutrophils that migrated from the central cell loading channel into the side migration channels.	None	0.83+/−1.6	14.9+/−22.37
			fMLP	110.5+/−25.8	62.7+/−86.6
			LTB4	139+/−49.6	73.0+/−76.4
2. Average Velocity	**V**	The average velocity of neutrophil chemotaxis in straight channels or the linear portion of the channels past the mazes [µm/min]	None	0.3+/−0.8	6.3+/−8.0
			fMLP	17.5+/−2.1	9.5+/−7.6
			LTB4	23.5+/−5.3 •	10.0+/−7.5 •
3. Directionality	**D**	The fraction of neutrophils that navigate through the mazes along the shortest route, without turning back at the bifurcations or being delayed at posts.	None	0+/−0 •	0.26+/−0.32 •
			fMLP	0.78+/−0.11	0.48+/−0.35
			LTB4	0.87+/−0.08	0.63+/−0.35
4. Average Migration Length	**TL**	The average distance that neutrophils migrate along the straight channel [µm].	None	0+/−0 •	22.7+/−11.7 •
			fMLP	425.6+/−45.1 •	29.3+/−10.6 •
			LTB4	469.2+/−22.4 •	31.1+/−12.9 •
5. Oscillatory Migration	**OM**	The percentage of neutrophils moving inside the channels that cross-back and forth over the mid-line (x = 250 µm) of the straight channel.	None	0+/−0	2.4+/−7.3
			fMLP	2.3+/−5.7	6.8+/−10.4
			LTB4	4.2+/−10.2	2.5+/−5.4
6. Through	**T**	The percentage of neutrophils that migrate persistently the entire length of the straight channels in the field of view (x = 500 µm).	None	0+/−0 •	11.64+/−19.8 •
			fMLP	88+/−6.3 •	14.0+/−21.4 •
			LTB4	93+/−8 •	31.2+/−33.9 •

Averages +/− standard deviations are shown for all parameters for both healthy donors and burn patients. • indicates average parameters that are significantly different between healthy donors and burn patients, (p<0.05)

### Neutrophil spontaneous migration is characteristic for sepsis

**Figure 3:**
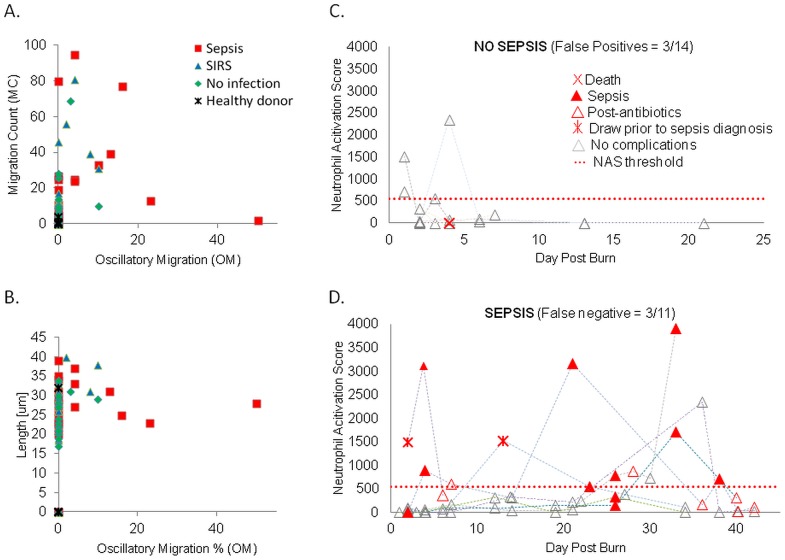
The components and temporal evolution of neutrophil spontaneous migration score (NAS_N_) in patients with major burns. (A) Two dimensional comparisons between neutrophil migration parameters between healthy donors (black stars), burn patients with no complications (green square), SIRS (green triangle), and sepsis (red square). In the absence of chemoattractant, neutrophils from patients with sepsis migrate in larger numbers and display more oscillatory migration than the patients with SIRS or those from healthy donors (which do not migrate). (B) Neutrophils from patients with sepsis migrate longer distances and display more oscillatory migration than the patients with SIRS or those from healthy donors (which do not migrate). (C) Changes in the neutrophil activation score in the 6 patients with no sepsis during the hospital stay. On patient that died is indicated (red cross). (D) Changes in the neutrophil activation score in the 7 patients that experience sepsis during the hospital stay. Higher scores are observed during sepsis (filled red triangle). In patients that respond to antibiotic treatment, NAS_N_ decreases when sepsis is resolved (empty red triangles). In some of the patients, NAS_N_ increased even several days before sepsis was diagnosed (red star). The NAS_N_ remained low in these patients in the absence of complications (empty gray triangle).

We compared the migration parameters of neutrophils from burn patients based on the status of the patient at the time of sample collection. Of the 71 blood samples from burn patients analyzed, 21 were collected from patients during sepsis, 29 during SIRS, and 21 from patients with no signs of SIRS. We observed that a significant numbers of neutrophils from patients during sepsis advanced inside the channels in the absence of a chemoattractant (**S3 Movie**). To better characterize this unique phenotype, we recorded the number of neutrophil migrating spontaneously (MC_N_) and the fraction of neutrophils moving spontaneously and crossing back and forth the longitudinal-middle of the channels (OM_N_ – **Fig. 2C**). However, on a two dimensional plot of oscillatory migration and migration count, the neutrophils samples from patients during sepsis often overlap with those in the absence of sepsis (**Fig. 3A**). Similarly, significant overlap between sepsis and non-sepsis samples was present when we analyzed the average distance that neutrophils advanced inside the channels length (TL_N_) vs. the oscillatory migration. Other parameters of neutrophil migration in the absence of chemoattractant did not correlate with sepsis, alone or in combinations (**Fig. 3B**). Moreover, no linear combination of the 18 parameters appeared to be effective in differentiating between sepsis and no-sepsis.

To better characterize the unique phenotype of spontaneous migration of neutrophils, we took into account the observation that these neutrophils either migrate long distances inside the channels or display oscillatory migration patterns. Thus, we defined a nonlinear neutrophil activation score in the absence of chemoattractant (NAS_N_), which captures both behaviors in the same equation: NAS_N_  =  MC_N *_(TL_N_ + OM_N_). We analyzed the changes in NAS_N_ during time after major burns and observed that the score remained low for the duration of hospital stay in patients with no sepsis (**Fig. 3C**, N = 28 samples from 6 patients). In contrast, in patients with sepsis, we observed transient increases of the NAS_N_ score that corresponded with the time of sepsis (**Fig. 3D**, N = 43 samples from 7 patients). The increases of NAS_N_ were usually transient during the first week after burn, and appeared to be more prolonged later in the disease (after week 3). Moreover, the score usually decreased after the start of antibiotic treatment and, in some of the patients that developed sepsis, the score increased days before sepsis was diagnosed (**Fig. 3D**).

**Figure 4:**
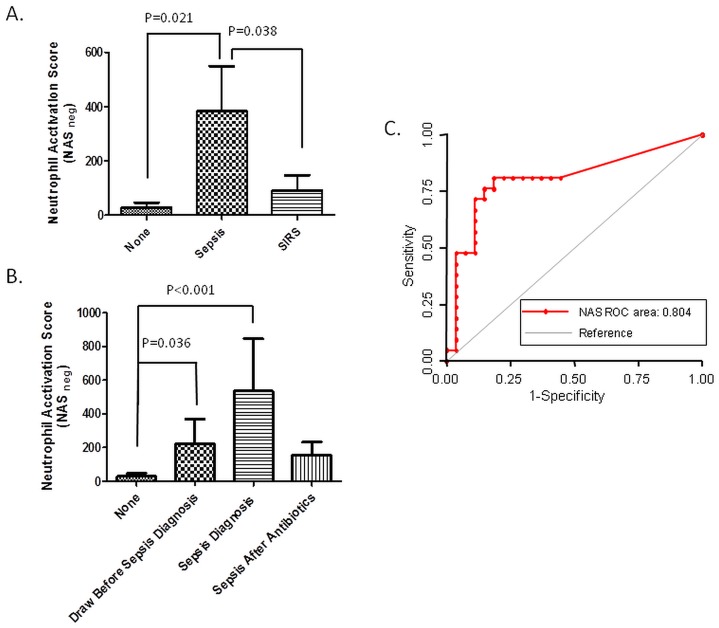
Correlations between the changes in neutrophil spontaneous migration score (NAS_N_) and the timing of sepsis in burn patients. (A) Neutrophil activation score averages are significantly different between burn patients with sepsis (N = 21) and patients with SIRS (N = 29, P = 0.038) or patients with no SIRS (N = 21, P = 0.027). (B) The NAS_N_ is significantly higher in patients in samples before clinical diagnosis (P = 0.036) and drops to one third following septic diagnosis and post-antibiotic therapy. (C) ROC curves for averaged NAS_N_ of two successive blood samples demonstrates the good predictive values of the spontaneous neutrophil migration patterns for sepsis in patients with major burns.

A logistic model of generalized estimating equations (GEE) indicated that the NAS_N_ score is statistically significant correlated with sepsis and differentiates between septic patients and those with systemic inflammatory response syndrome (SIRS - P = 0.038) and no complications (P = 0.021, **Fig. 4A**). The score increased significantly at least 3 days before clinical diagnostic of sepsis (P = 0.036). Moreover, the NAS_N_ decreased 3-fold after effective antibiotic treatment was started, compared to the first draw after sepsis diagnosis (**Fig. 4B**). A random intercept linear mixed effects model (repeated measures of ANOVA) revealed that the mean of the NAS_N_ during infection was different than that of the infection-free state (p = 0.077).

To further probe the potential utility of analyzing the spontaneous neutrophil migration patterns that act as a biomarker for sepsis in burn patients, we analyzed the receiver operating characteristic (ROC) for various cut-off levels of NAS_N_. Using the NAS_N_ on the day of the sepsis the area under the curve was 0.74, at the threshold of good performance. Moreover, the performance of the NAS_N_ could be improved further by considering the average of two successive samples. The area under the curve increases to 0.80, the best sensitivity to 80%, and specificity to 77% (**Fig. 4C**). This analysis suggests that the NAS_N_ has the potential, when monitored frequently, to become useful adjunct to current clinical protocols to identify and monitor sepsis in patients after major burns.

## Discussion

The microfluidic platform presented in this study enabled us to characterize the migration of human neutrophils from burn patients, at single-cell resolution and with high precision, across 18 parameters. In addition to confirming that significant differences in neutrophil migration phenotype exist between burn patients and healthy [40], we uncovered a new neutrophil migration phenotype, the spontaneous migration in the absence of chemoattractants, which is highly correlated with the occurrence of sepsis in the burn patients. The spontaneous migration of neutrophils is a unique phenotype, which has not been observed before. The spontaneous migration takes place in the absence of any chemoattractants and is thus distinct from *chemokinesis*, which requires continuous exposure to chemoattractant. The spontaneous migration shows long strides of persistent migration, however, it is different from *chemotaxis* because these strides occur in the absence of any chemical gradient to trigger the persistent migration. Spontaneous migration is also unlike *retrotaxis*, when neutrophils move persistently and independently against chemical gradients, but only following chemotaxis [41].

The spontaneous neutrophil migration phenotype could not be captured by existing chemotaxis assays. Conventional assays, such as the transwell assay [21], do not have single cell resolution. They only measure average migration, and will miss entirely a small population of cells with spontaneous and oscillatory migration phenotype. Other assays, recently reviewed in [25], [42] lack the necessary precision to characterize complex neutrophil migration patterns like those during spontaneous cell migration. Most of microfluidic devices where neutrophils migrate on one-dimensional surfaces [22], [26], [29], could not decouple the oscillatory migration from changes in direction, and thus do not have the necessary precision to distinguish the new phenotype. Moreover, the new microfluidic platform is robust, easy to use, and requires no external pumps or other accessories besides the microscope.

Overall, sensitivity and specificity values for our assay are only slightly better than other markers for sepsis in burn patients [43], [44]. However, it is important to note that, enabled by technology, we could observe the changes in neutrophil migration phenotype, up to two days before sepsis. This unique capability of our assay, to predict the occurrence of sepsis, may eventually enable early interventions or preventive treatments in the context of burns. It also points to a potential critical role that neutrophils play in the earliest stages leading to sepsis. The spontaneous neutrophil migration phenotype may be an integrator of multiple inputs (cytokines, chemokines, leukotrienes, extracellular matrix context, and microbial products), which allows neutrophils to prioritize these signals for their contextual importance [45] during tissue injury [46] and infections [47]. Until now, such stimuli could only be on measured individually e.g. CD64 [11], IL-6 [48], serum macrophage migration inhibitory factor [49], interleukin-1 receptors [50], and neutrophil gelatinase-associated lipocalin [51] and integrated into comprehensive scores afterwards by artificial models. Using the neutrophil phenotype as a marker has the advantage of natural adaptation to changes and compensation for variability in the levels of signals among individuals.

Additional benefits from the use of microfluidic technologies to monitor neutrophil function may eventually emerge, besides the better precision for identifying sepsis. Correcting the functionality of neutrophils in patients could become as an important objective among sepsis prevention measures. Supporting this possibility, one recent study showed that treatment using lipid mediators of inflammation resolution could help restore normal neutrophil migration phenotype and improve the survival after burns in an animal model [39]. A reliable infection marker in the context of burns and treatments to correct neutrophil functionality could discourage the preventive use of antibiotics and reduce the chances of selecting for antibiotic resistant bacterial strains, which are common in burn units [52]. Further studies of neutrophil phenotype in larger populations of patients with sepsis after major burns could lead to better understanding of the roles that neutrophils play before and during sepsis and facilitate the design of new tools for the diagnosis, monitoring, and prevention of sepsis in patients with major burns.

## Supporting Information

S1 Movie **Neutrophils from healthy volunteers migrate fast and efficiently around the posts in the channel.**
(AVI)

S2 Movie **Neutrophils from patients with major burn injury display defective migration.** Patient neutrophils often get stuck on the posts, lose directionality, and reverse the direction of migration to exit the migration channel.
(AVI)

S3 Movie **A significant numbers of neutrophils from patients during sepsis advanced inside the channels in the absence of a chemoattractant.**
(AVI)
